# Nutrition Behaviors in Polish Adults before and during COVID-19 Lockdown

**DOI:** 10.3390/nu12103084

**Published:** 2020-10-10

**Authors:** Ewa Błaszczyk-Bębenek, Paweł Jagielski, Izabela Bolesławska, Anna Jagielska, Aneta Nitsch-Osuch, Paweł Kawalec

**Affiliations:** 1 Department of Nutrition and Drug Research, Institute of Public Health, Faculty of Health Sciences, Jagiellonian University Medical College, 20 Grzegórzecka Str., 31-531 Krakow, Poland; paweljan.jagielski@uj.edu.pl (P.J.); pawel.kawalec@uj.edu.pl (P.K.); 2 Department of Bromatology, Poznan University of Medical Sciences, 42 Marcelińska Str., 60-354 Poznań, Poland; ibolesla@ump.edu.pl; 3 Department of Social Medicine and Public Health, Medical University of Warsaw, 3 Oczki Str., 02-007 Warsaw, Poland; anna.jagielska@wum.edu.pl (A.J.); anitsch@wum.edu.pl (A.N.-O.)

**Keywords:** COVID-19, eating habits, lockdown, social distancing, food consumption

## Abstract

Unexpected isolation, which has not yet been seen on a global scale, has created the conditions for evaluating nutrition in a situation of reduced spatial activity. The study aimed to assess the influence of lockdown on selected eating habits of Polish adults. An anonymous questionnaire was conducted, including questions about eating habits and self-reported anthropometric measurements, referring to “before” and “during” lockdown. We reported the findings of 312 adults (aged 41.12 ± 13.05 years). Overall, 64.1% of the participants were women, 77.7% urban inhabitants and 78.6% employed. The average length of social isolation was 50.79 ± 10.53 days. The majority (51.6%) of the respondents did not eat outside the house during lockdown (*p* < 0.0001). The number of meals eaten during the day during lockdown increased significantly, 11.2% of the respondents ate 5 and more meals (*p <* 0.0001). The percentage of people snacking between meals increased by 5.1% during lockdown (*p =* 0.0001). Eggs, potatoes, sweets, canned meat and alcohol were consumed considerably more commonly during lockdown, while fast-food products, instant soups and energy drinks were eaten or drunk significantly less frequently. A marked decrease in the number of daily servings of the following products was observed: bakery products, red meat, fast food, instant soups, sweet beverages and energy drinks. Conversely, the number of daily servings of sweets and canned meat significantly increased. Two thirds of the respondents reported body weight changes, with 45.86% of the participants being overweight during lockdown. Significant changes in the diet of Polish adults were found during lockdown due to COVID-19.

## 1. Introduction

The Minister of Health announced the state of the epidemic in Poland on 20 March 2020, which resulted in closing borders and imposing entry restrictions on travelers. Before the epidemic was announced, educational activities in school and college settings had been suspended since 12 March. The parents of children aged up to 8 or ill children aged up to 14 years were entitled to an additional care allowance due to COVID-19 related restrictions (claimed until 14 June) and stayed with the children at home [1,2]. Numerous announced restrictions (bans on public meetings, closed parks and forests, shopping mall restrictions, closed restaurants with only take-away meals or door-to-door food deliveries available) lasted until May 2020 [3]. By the second half of September, almost 80,000 cases of COVID-19 in Poland had been reported, and nearly 2298 people had died [4].

Social distancing influenced both free-time and occupational physical activity. According to the report, 17% of Poles worked at home during the COVID-19 pandemic. Moreover, according to one report 4% of Polish parents received additional care allowance while taking care of children [5]. Potentially, those restrictions and the mandatory quarantine (over 100,000 people have been obligatorily quarantined for 14 days since the beginning of the epidemic in Poland) of many people in Poland influenced their nutritional status and eating habits [3]. According to published scientific results, nutritional habits and physical activity are recognized as the essential factors of Body Mass Index (BMI, kg/m^2^) and health status [6,7,8].

Food safety was one of the most important challenges during the pandemic. According to the European Food Safety Authority (EFSA), no evidence was provided to confirm that food was a likely source or the route of transmission of the virus [9]. Particular principles concerning food processing and information on food safety were also found among Polish recommendations [10,11]. Furthermore, food safety, ensuring continuous deliveries of fresh products, was at a safe level [12]. However, the fear of the disruption of deliveries made Poles buy large amounts of hygienic products and products with long use-by dates [13]. Due to the reduction in trade in traditional shops in April 2020 compared to the previous month, a significant increase in retail sales via the Internet (by 27.7%) was recorded. It was noted mostly in industrial products, but also in food (14.9%) [14].

Nutrition exerts a significant impact on overall health and the risk of disease. In relation to infectious diseases such as influenza and the new SARS-Cov-2 virus, food and nutrients also influence the way our immune system functions [15]. In this specific period, as preliminary studies by other authors showed, self-isolation may have been associated with a change in selected eating habits, including snacking or overeating caused by staying at home or lower physical activity that changed energy requirements [16,17]. The studies published so far, both from Poland [17,18] and internationally, highlighted the impact of the pandemic on dietary behaviors and the need for some changes in connection with building up resistance [15]. The WHO/Europe recommendations also constitute a response to those studies that showed the need to pay special attention to dietary behaviors, including portion sizes, which was also shown in this publication [19]. Therefore, the aim of the study was to describe the potential effect of social isolation on the eating habits and nutritional status of adult people in Poland.

## 2. Materials and Methods

The observational retrospective online questionnaire study using social media to distribute the anonymous online questionnaire was conducted from 29 April until 19 May 2020. The inclusion criteria were age over 18 and the ability to give informed consent to participate in the study. The exclusion criteria were no consent to participate in the study, age < 18, pregnancy, coexisting diseases requiring a specific diet. The response rate was 48.1%. Healthy adults were the target population.

All the participants were fully informed about the study requirements and were required to accept the data sharing and privacy policy prior to the study. It was emphasized that the participation in the study was voluntary and all the participants accepted a patient information form with the presentation of the study. Information on informed consent to participate in the study was on the first sheet of the online questionnaire, on which the respondent also gave consent. The study was conducted in accordance with the principles of medical research ethics contained in the Helsinki Declaration. The research protocol was submitted to and acknowledged by the Ethical Committee of the Medical University of Warsaw (AKBE/122/2020). A completed questionnaire was transmitted to the Jagiellonian University questionnaire platform and the final database was downloaded as a Microsoft Excel sheet.

### 2.1. Survey Questionnaires

The survey included 96 questions based on the Dietary Habits and Nutrition Beliefs Questionnaire for people aged 15–65, designed by the Behavioral Nutrition Team, Committee of Human Nutrition, Polish Academy of Sciences, validated and recommended for research in the Polish population [20]. The part of the questionnaire that was used in our survey referred to dietary habits and included the following questions: How many meals do you usually consume daily? Do you consume meals at regular times? How often do you snack between the meals? What types of food do you usually consume between the meals during the weekdays? Food frequency consumption for 26 products and 7 beverages—with the following responses and scores: (1) never, (2) 1–3 times a month, (3) once a week, (4) few times a week, (5) once a day, (6) few times a day [20]. In the present authors’ section, we also asked about the number of portions of a habitually eaten product, using home measurements for the determination of the size of portions for each product and/or photographs showing the size of portions. The responses and scores for portion sizes were as follows: (1) zero, (2) half a portion, (3) one, (4) two, (5) three, (6) four or more. One portion size for each product was determined. For bakery products it was 1 slice weighing 20 g and for wholemeal bread—1 slice weighing 30 g; white rice or white pasta and groats, buckwheat, oats—half a glass of boiled rice, pasta and groats; butter, lard and oils or margarines—5 g, a small spoon; milk and fermented milk—a 200 mL glass; fresh cheese—a slice of cheese weighing 30 g; cheese—2 slices weighing 10 g each; cold meats—20 g; red and white meat, fish—100 g; eggs—one egg of 50 g; pulses—half a glass of boiled product; potato—100 g—two small potatoes about 5 cm in diameter; fruits—100 g, e.g., one small apple, or one small pear, or five plums; vegetables—100 g, e.g., two small tomatoes 5 cm in diameter, or one small pepper; fast food and fried foods—100 g; sweets—50 g, e.g., half a bar of chocolate; instant soups—a 300 mL plate; tinned meats and vegetables—100 g. As regards beverages the following portion sizes were assumed: fruit, vegetable and mixed juices as well as hot beverages, like tea or coffee—a 200 mL glass; sweetened carbonated or non-carbonated soft drinks, energy drinks, mineral water—a 200 mL glass. One portion of alcoholic beverages meant half a glass, i.e., 100 mL. The same questions were asked twice and referred to the time before and during the social isolation with reference to the announcement of the state of the epidemic in Poland.

The demographic data included age, gender (man, woman), place of residence (a village, a town below 20,000 inhabitants, a town between 20,000 and 100,000 inhabitants, a city over 100,000 inhabitants), the number of people in the household, financial situation in relation to the epidemic situation (below average, average, above average), occupation (no, I am retired or receiving a disability living allowance; no, I am on maternity leave; I am unemployed or other (a housewife/househusband); yes, but it is only a temporary job; yes, I am permanently employed; no, I study), education level (primary, lower secondary, upper secondary, higher) [20]. Remote work before epidemic: (1) no; (2) yes, partly; (3) yes, I work from home, and during epidemic: (1) yes, I work as before; (2) yes, I have been working remotely for … (a number of days); (3) no, I’m taking care of a child/children under 8; (4) no, I’m on an unpaid leave. The last question referred to the self-assessment of eating habits: In your opinion, has your diet changed due to the epidemiological situation and limited activity? (1) No, (2) yes, I eat more, (3) yes, I eat less. The respondents also answered a question about the use of dietary supplements—yes (1), no (0). If a supplement was used, they entered the name, type and dose of the supplement. On this basis, dietary supplements were classified into 9 groups.

The participants provided information concerning the following anthropometric measurements including weight (kg), height (cm), waistline (cm)—before and during lockdown (height was noted only once). Based on the reported measurements, BMI value was calculated for each participant at two time points—before and during social isolation. The interpretation of the patients’ nutritional status on the basis of the BMI was made in accordance with the WHO guidelines for adults [21].

### 2.2. Statistical Analysis

The results for age and anthropometric data were presented as the mean value of ±SD and the median. Other variables were presented as a percentage of responses. In order to check the differences for the tested variables before and during the isolation, the Wilcoxon test or the McNemar–Bowker test were applied. The analysis of data was carried out in PS IMAGO PRO 6 (IBM SPSS Statistics 26, Chicago, IL, USA), and the level of statistical significance was assumed at *p <* 0.05.

## 3. Results

### 3.1. Study Group Baseline Characteristics

A total of 312 participants from 15 (out of 16) voivodeships from Poland completed the online questionnaire and were included in the analysis. The average length of the social isolation of the whole study group was 50.79 ± 10.53 days. Overall, 64.1% of the participants were women. The group included 77.7% of urban inhabitants, and 78.6% of all participants were employed. The average age of the participants was 41.12 ± 13.05 (male: 42.11 ± 12.04 years, female: 40.58 ± 13.57 years; *p =* 0.2350). The demographic characteristics of the participants are shown in Table 1.

Remote work had been performed by 3.2% of the study group before social isolation started. Of the participants, 21.7% had performed partial remote work, and 75.1% had not worked remotely. During social isolation, almost 66.6% of the study group worked remotely, 4.2% were on care allowance, and 7.8% were on leave. Social isolation influenced the financial situation of 33.1% of the participants, including an improvement in 5.2% and deterioration in 27.9% of them.

### 3.2. The Nutritional Status of the Study Group before and during Confinement

The average body weight (kg) of the whole group before the confinement had been 73.47 ± 16.65 kg, while during the confinement it was 74.03 ± 16.81 kg (Δ 0.56 ± 2.43 kg; *p <* 0.0001). Only 106 respondents knew their waist circumference before lockdown and it was 87.12 ± 15.58 cm. Waist circumference during confinement was reported by 201 participants: 87.96 ± 14.26 cm. Statistically significant differences were observed before (24.98 ± 4.33 kg/m^2^) and during (25.28 ± 4.44 kg/m^2^; *p <* 0.0001) confinement as regards the nutritional status based on BMI (Δ 0.27 ± 1.61 kg/m^2^). Data concerning the nutritional assessment of the study group based on the interpretation of BMI before and during lockdown are presented in Table 2.

**Figure 1:**
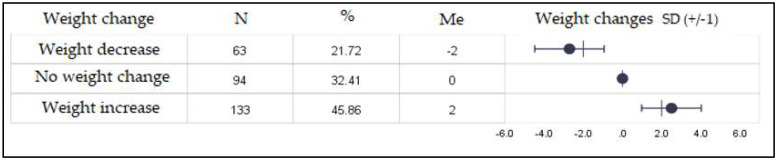
Changes in the body weight of the study group (%). *N*—number of participants; Me—median; SD—standard deviation.

Figure 1 (below) presents changes in the body weight of the study group. Body weight changed in over 2/3 of the participants, with 45.86% of the participants being overweight during lockdown.

### 3.3. Eating Habits of the Study Group before and during Confinement

The self-assessment of the changes in the diet of the respondents during lockdown showed that most of the study group did not introduce any changes into their dietary habits during lockdown (32.4%; Table 3).

**Figure 2:**
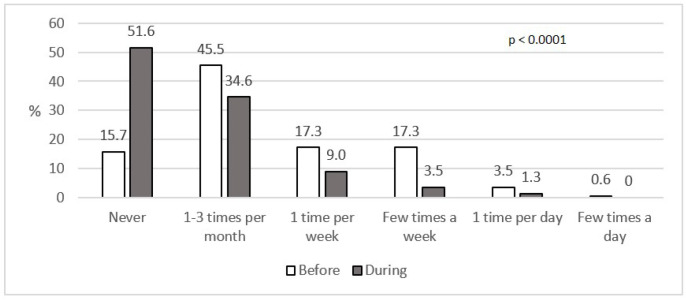
Eating out and ordering take-away food, before and during social isolation (%).

A significant increase (*p <* 0.0001) was observed in the number of people who did not eat outside the house, but also did not order take-away food during social isolation. Figure 2 below shows that during lockdown 51.6% of the participants did not eat outside or did not order take-away food compared to 15.7% before lockdown (Figure 2).

**Figure 3:**
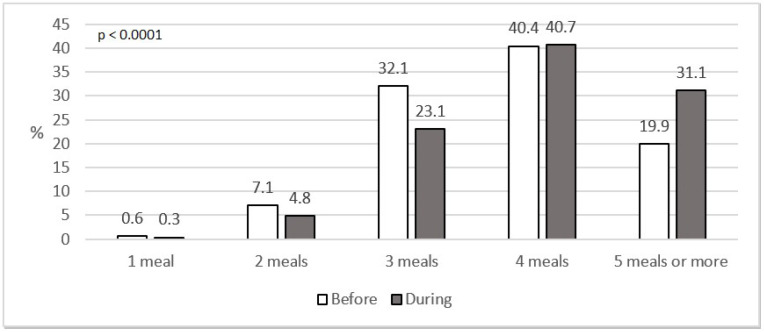
The number of meals eaten daily before and during social isolation (%).

Figure 3 presents the number of meals before and during social isolation. The respondents differed as regards the number of meals consumed before and during the COVID-19 pandemic (*p <* 0.0001). The increase of 11.2% was noted in the group of people who ate 5 or more meals per day.

Over half of the participants ate some of their meals before (53.2%) and during (51.9%) lockdown regularly. Meals were always consumed regularly by 27.9% of the participants before and 33.7% during confinement (*p =* 0.1002). Of the respondents, 72.8% reported that before the pandemic they had snacked regularly (few times per week and more). The respective percentage during the pandemic was 77.9% (*p =* 0.0001). Regardless of the time of the survey, the most commonly selected snacks in the study group were fruits (before 63.8%, during 64.7%, *p =* 0.7755); sweet snacks, e.g., confectionary, biscuits, cakes, chocolate bars, cereal bars, wafers (51.9% before and during the pandemic, *p =* 1.0000) and nuts, almonds, seeds (before: 44.6% and during lockdown: 43.6%, *p =* 0.7838). Salty snacks such as crackers, pretzels, crisps, potato chips and fries were eaten more frequently as snacks during lockdown than before (before 26.3%, during 31.4%, *p =* 0.0386).

Vegetables were the most frequently eaten by the whole study group. They were chosen several times per day by 27.2% before and 26.9% of the participants during confinement (*p =* 0.4241). The second most commonly selected product was butter, used as a bread spread or as an addition to meals/for frying/for baking, etc. by 25.6% before and 22.1% during confinement (*p =* 0.7000). Furthermore, bakery products, e.g., wheat bread, rye bread, wheat/rye bread, toast bread, bread rolls, were frequently eaten by 20.5% before and during confinement (*p =* 0.3737). The lowest food frequency consumption (understood as “few times per day”) was observed in case of red meat, e.g., pork, beef, veal, mutton, lamb, game (0.0% before and 0.3% during confinement; *p =* 0.1745), pulse-based foods, e.g., with beans, peas, soybeans, lentils (0.0% before and 0.3% during confinement; *p =* 0.7694). When the restrictions were imposed, the respondents changed the frequency of consuming products such as eggs (*p =* 0.0022), potatoes (*p =* 0.0004), sweets (*p =* 0.0241) and canned meat (*p =* 0.0004), which were eaten more frequently during lockdown. Fast foods and instant soups were consumed less frequently by the study group during lockdown (*p =* 0.0001 and *p =* 0.0247, respectively). Table 4 presents the exact rates of the consumption of selected products before and during lockdown.

Hot beverages, such as black tea, coffee, herbal or fruit teas, were chosen the most frequently (a few times a day)—by 72.4% before and 70.2% of the respondents during lockdown (*p =* 0.1500). Mineral water was the second type of beverages selected most commonly in both groups depending on the time point of the research (66.0% before and 69.6% during lockdown, *p =* 0.6431). Changes in the frequency of drinking selected beverages before and during the restrictions concerned energy drinks (*p =* 0.0150), which were drunk less frequently, and alcohols, which were drunk more frequently, during social isolation (*p =* 0.0031). Table 5 presents the exact consumption of selected beverages before and during the restrictions.

Vegetables constituted the largest number of portions. The average consumption was 200 g per day, regardless of the period studied. Moreover, the number of portions of bakery products, e.g., wheat, rye, mixed wheat-rye, toast bread, rolls, croissants was higher in the study group. The number of the daily servings of bakery products (*p =* 0.0400), red meats (*p =* 0.0199), fast foods (*p <* 0.0001) and instant soups (*p =* 0.0283) significantly decreased over the analyzed time intervals. However, the number of the daily servings of sweets (*p =* 0.0029) and canned meat (*p =* 0.0390) increased (Table 6).

Hot beverages, such as tea, coffee, herbal or fruit infusions (about 757 mL per day on average) and mineral water, the consumption of which was reported to be 767 mL per day (considering the number of portions), constituted the largest portion of beverages in the study group. A decrease was observed in the number of daily portions of two types of beverages consumed during lockdown compared to the time before lockdown: sweet drinks (*p =* 0.0254) and energy drinks (*p =* 0.0008) (Table 7).

**Figure 4:**
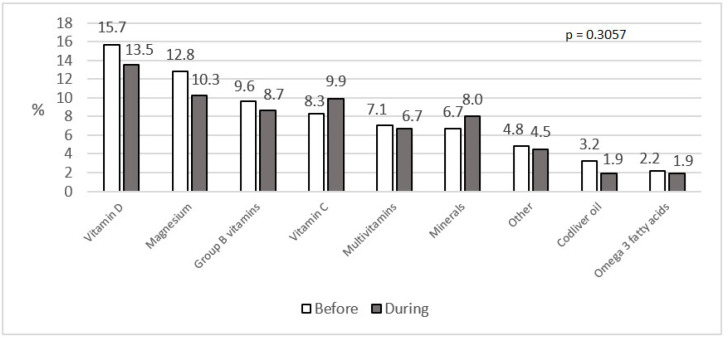
Dietary supplements before and during social isolation (%).

No significant differences in using dietary supplements (*p =* 0.3057) were noted between two points of time in the study group. Before lockdown 36.5% of the participants had used supplements, and 33.7% used them during lockdown. The dietary supplement used most commonly in the study group was vitamin D (15.7% before and 13.5% during confinement; *p =* 0.2810). Magnesium was used the least frequently (2.2% before and 1.9% during confinement; *p =* 0.4545). The distribution of dietary supplement use among respondents before and during the lockdown is presented in Figure 4.

### 3.4. Limitations

One of the aims of the present study was to illustrate as accurately as possible the potential changes associated with the lockdown, not only by the frequency, but also by determining the portion sizes of the food eaten. Despite the large workload, there are some limitations of the presented study, which should be considered when evaluating the results. The average weekly or daily changes of food consumption during the first weeks or days of lockdown could lead to the potential overestimation and limitation of this study. The study included retrospective data concerning the time before lockdown, based on the respondents’ memory, which may affect the presented eating habits. The research tool, which was the electronic questionnaire, was more often completed by respondents with higher education and from larger cities, probably also due to the better quality of Internet connections. The questionnaire itself was quite long, as it included almost 100 questions relating to two timeframes. Another limitation of the study was related to the determination of the portion size, because the products within the groups differed greatly. Therefore, it was not possible to use home measurements or the photographs of products that made it easier to determine the portion size for the subjects. However, it is certainly a preferable solution, despite some limitations.

## 4. Discussion

In the light of the COVID-19 pandemic, staying at home was recommended as the basic way to limit human exposure to the spread of the virus in Poland and in other countries [22,23,24]. It also resulted in changes in food shopping behaviors. Polish consumers bought mainly packed, dry food products, such as rice, flour and pasta, followed by tea, coffee, canned food, raw or cured meat, and bottled water, while fresh products (like fruits and vegetables) or sweets were less popular [25]. The changes in the types of purchased food products did not influence the observed eating habits in the Polish adult population, as reported in our study or by Sidor and Rzymski [18].

The present study mainly included middle-aged people from large cities, with a higher education level, most of whom (2/3 of the respondents) worked remotely from home due to the lockdown. The average time spent in social isolation was 51 days in the study group. The body weight of 32.41% of the respondents did not change during social isolation, while the weight of as much as 45.86% of the respondents significantly increased (Figure 1). Several factors, such as those related to lifestyle, like physical activity and eating habits, may have influenced the change of body weight. Sidor and Rzymski conducted a survey in a group of young people (the average age: 27.7 ± 9.0), with the majority of them being women (95.1%). They reported that almost 40% of them observed no changes in their body weight. In the same Polish study, weight changes were correlated with such factors as age (r_s_ = 0.15, *p <* 0.05), which was particularly visible in the age groups 35–45 and >45 years [18]. The outcomes reflected the results of the present research.

No changes in the diet were reported by the respondents (32.4%) in the self-assessment of their diet over the analyzed time intervals. Sidor and Rzymski reported that 43.5% of the respondents declared eating more [18]. The distribution of changes in people’s eating habits due to lockdown in Great Britain from April 2020 showed that 40% ate slightly or much more. The same 47% of almost 2000 respondents were at the age of 18 and more. According to the respondents’ opinion, 20% of them demonstrated healthier eating habits than before [26]. It means, however, that in case of the other participants of the present study, some changes were noted as regards the frequency of the consumption of selected products and beverages, and/or the number of the portions of products eaten during the day. As the results showed, the most frequently indicated change in nutrition was an increase in the number of portions (18.3% of the respondents) while maintaining the same product selection (Table 3).

The frequency of eating outside the house or ordering meals at home was among the examined aspects of the diet before and during lockdown. Regarding the present respondents, as many as 51.6% did not eat outside the house and did not order food during lockdown, while before social isolation, over 45.5% had eaten in this way (*p <* 0.0001). An analysis performed by the United States Department of Agriculture showed that food consumed away from home was a contributing factor to poor diet quality and obesity [27]. In the light of the recommendations of the WHO, eating at home reduced the rate of contact with other people and lowered the chance of being exposed to COVID-19 [28].

Lockdown had a significant impact on the total number of meals consumed in the study group with the number increasing significantly (*p <* 0.0001). The percentage of people eating 5 meals per day increased from 19.9% to 31.1% (Figure 3). The findings are similar to those of other researchers [18,29]. According to the literature, eating frequency was positively associated with reductions in fat mass and body fat percentage as well as an increase in fat-free mass [30].

The time spent at home during social isolation was also significantly correlated with snacking (*p =* 0.0001). Before the pandemic, 72.8% of the respondents had snacked regularly (several times per week and more). During the pandemic, the respective percentage was 77.9%. During the COVID-19 pandemic, the respondents chose salty snacks like crackers, pretzels, crisps, potato chips or fries (*p =* 0.0479) more often than before. An international online study included a thousand respondents, with 54% of them being women. It showed that the number of snacks between meals or late-night snacking increased significantly during home confinement (*p <* 0.001) [29]. In the previously mentioned study by Sidor and Rzymski, overweight (55.3%) and obese (61.7%) respondents snacked significantly more commonly [18].

The results of this survey also revealed that the changes in the frequency and number of products consumed did not only include those that favored shaping eating habits but also those that promoted health and the maintenance of healthy body weight (Table 4, Table 5, Table 6 and Table 7). The frequency of the consumption of products such as eggs (*p =* 0.0022), potatoes (*p =* 0.0004), sweets (*p =* 0.0241) and tinned meat (*p =* 0.0004) increased significantly during lockdown. In patients with type 2 diabetes from India, data from a survey conducted at a similar time (45 days after the start of lockdown) showed that carbohydrate consumption and the frequency of fruit consumption increased in 21% and 27% of study participants, respectively [31]. Pietrobelli et al. studied a sample of 41 children and adolescents with obesity from Verona, Italy, and reported no changes in vegetable intake and increased fruit intake (*p* = 0.055) during lockdown. At the same time, the consumption of potato chips, red meat, and sweetened beverages significantly increased [32].

In Poland, food was widely available, except for temporary deficiencies in bakery yeast or ready-to-eat products with long shelf life. However, with the exception of canned meat, we have not observed any increase in the consumption of this category of products in our results. No significant increase was observed in the consumption of products with long expiry date, which were most willingly bought in the first days of social isolation in Poland. Moreover, we did not observe any increase in the consumption of health-promoting products recommended by the Food and Nutrition Institute (currently a part of the National Institute of Public Health, National Institute of Hygiene) such as vegetables and fruit or pickled food, low-fat dairy products [33]. Consumer behavior evaluated in a Tunisian study showed that 89% of the respondents declared to be aware of food waste, and the COVID-19 lockdown would impact 93% of them; their waste levels; and, for 80%, their grocery shopping habits [34]. Significant reductions in the frequency of the consumption of fast-food products (*p =* 0.0001) and instant soups or ready-made jars (*p =* 0.0247) were among the positive changes observed due to lockdown. Changes in the number of consumed portions were visible such as the consumption of a smaller number of portions of wheat bread (*p =* 0.0400) and red meat (*p =* 0.0199). Similarly, the frequency of the daily consumption of selected fast-food products (*p <* 0.0001) and instant soups (*p =* 0.0283) significantly decreased. An increase in the frequency of the consumption of products such as sweets and meat preparations was accompanied by a simultaneous increase in the number of portions consumed by the subjects during lockdown (*p <* 0.05). An Italian survey showed an increase in homemade Italian recipes, cereals, legumes, white meat and a decrease in fresh fish, packaged sweets, baked products, and delivery food [35].

As regards drinks consumed during lockdown, the consumption of energy drinks decreased significantly (*p =* 0.0150) in Poland. The consumption of alcoholic beverages significantly increased (*p =* 0.0031), which does not correspond to the results obtained by Italian researchers who reported a decrease in alcohol consumption [35]. However, it is consistent with previous studies confirming a high proportion of alcoholic beverages among all drunk fluids, especially in men [36]. A significant reduction in the number of the portions of energy drinks was a positive change observed in this study. It may be related to a partial abandonment of the obligation to work and/or the introduction of remote work, as well as a reduction in the number of consumed portions of sweet drinks (*p =* 0.0254).

WHO/Europe developed specific guidance for periods of self-quarantine because of the COVID-19 pandemic. Therefore, it is recommended to use fresh products, especially fruits and vegetables, and also to continue the consumption of reduced-fat dairy products. In our research, no significant increase was observed as regards the frequency of the consumption of fruit and vegetables, except potatoes. The results are also confirmed by the data collected by the Central Statistical Office in Poland, where a decrease in the consumption of vegetables (except potatoes) and fruit in households was observed [37]. Regrettably, our participants also did not follow the recommendations in the aspect of reducing the consumption of some products, like sweets (*p =* 0.0241) and canned meat (*p =* 0.0004) which were eaten more frequently during lockdown. With regard to WHO/Europe COVID-19 guidelines, the present study revealed positive changes in the reduction of eating out—51.6% of the participants did not eat outside the house or order take-away food compared to 15.7% before lockdown. Food delivery is one of the recommended options, but as we know, such food is richer in fat and sugar. Being aware of portion sizes, is, inter alia, one of the recommended points. Therefore, this study also focused on this aspect. It showed that despite the fact that the frequency of some products increased, portion sizes did not change, e.g., alcohol or white bread was not ingested more frequently but the number of daily servings increased [19].

According to Polish recommendations, vitamin D should be supplemented by adults (19–65 years old) from September to May [38]. However, in the study group only 15.7% applied this recommendation before and 13.5% during lockdown. Overall, regardless of the time point, the use of dietary supplements in the study group was lower than indicated by market data, referred to in a paper by Dziedziński et al., who reported that 72% of Poles took dietary supplements [39].

## 5. Conclusions

The results of the study indicated some changes that occurred in the study group due to the implementation of social isolation during the COVID-19 pandemic. The changes were observable in the number of meals consumed and the reduction in consumption, both—the frequency and the number of portions of products, such as white bread, red meat, ready-made soups, canned meat or fast-food products, as well as sweet and energy drinks—which constitute the positive changes in eating behaviors. However, we also noted an increase in the consumption of sweets and alcoholic beverages, which may be considered as not contributing to health and weight maintenance. No increase in the consumption of products with a long use-by date was observed. With some exceptions, the findings may suggest that, in the long term, nutrition behavior does not change during lockdown, nor does it increase the proportion of healthy products in the diet. In periods of reduced physical activity, e.g., during lockdown, we should eat less, especially high-calorie products, but more fruit and vegetables or low-fat protein products, which have a positive effect on building up resistance.

## Figures and Tables

**Table 1:** Demographic characteristics of the study group.

Variables	*N*	%
Age (years)		
≤24	27	8.7
25–65	263	84.2
≥66	22	7.1
Gender		
Female	200	64.1
Male	112	35.9
Place of living		
Village	69	22.3
Town <20,000 citizens	30	9.7
Town >20,000–100,000 citizens	48	15.5
City >100,000 citizens	162	52.5
Occupation		
Unemployed	11	3.6
Student	25	8.1
Employed	243	78.6
Retired	30	9.7
Level of education		
Primary	3	1
Vocational	7	2.3
Secondary	65	21.2
Higher	232	75.5

*N*—number of participants.

**Table 2:** Nutritional assessment interpretation basing on Body Mass Index (BMI) of the whole study group.

Nutritional Status	Before Confinement	During Confinement	*p*-Value
Underweight, *n* (%)	6 (2.1)	7 (2.3)	
Normal weight, *n* (%)	154 (53.3)	153 (50.8)	0.7213
Overweight, *n* (%)	94 (32.5)	101 (33.6)	
Obese, *n* (%)	35 (12.1)	40 (13.3)	

**Table 3:** Self-assessment of changes in the diet of the whole study group, *n* = 312.

Answers to the Question: In Your Opinion, Has Your Diet Changed Due to the Social Isolation?	*N*	%
No, I was eating the same kind and quantity of food.	101	32.4
Yes, I was eating the same products, but in greater quantities.	57	18.3
Yes, I was eating the same products, but in smaller quantities.	30	9.6
Yes, I have changed my product range without changing the quantity.	48	15.4
Yes, I have changed my product range and I eat less.	31	9.9
Yes, I have changed my product range and I eat more.	45	14.4

*N*—number of participants.

**Table 4:** The frequency of the consumption of selected products before and during lockdown (%).

Food Product	Time	Never	1–3 Times a Month	Once a Week	Few Times a Week	Once a Day	Few Times a Day	*p*-Value
White bread	B D	6.7 6.1	9.6 8.7	11.9 16.3	29.5 30.1	21.8 18.3	20.5 20.5	0.3737
Wholemeal bread	B D	16.3 20.5	22.1 13.1	11.2 16.0	25.6 28.8	16.7 14.7	8.0 6.7	0.5118
White rice, white pasta	B D	5.4 6.1	15.7 16.7	29.2 26.0	42.3 45.2	6.4 5.8	1.0 0.3	0.7314
Buckwheat, oats	B D	9.6 11.2	23.1 17.0	21.5 24.7	36.5 36.9	7.4 7.7	1.9 2.6	0.2765
Butter	B D	12.8 10.3	9.0 6.7	8.0 11.2	23.4 27.2	21.2 22.4	25.6 22.1	0.7000
Lard	B D	73.7 76.0	16.7 10.6	3.8 6.4	3.8 4.2	1.6 2.6	0.3 0.3	0.2404
Oils or margarines	B D	20.2 18.3	15.7 14.1	16.0 14.4	31.7 38.1	11.9 11.9	4.5 3.2	0.1060
Milk	B D	16.0 16.7	15.4 13.1	7.1 7.4	19.9 21.2	25.0 25.3	16.8 16.3	0.8312
Fermented milk	B D	9.9 11.2	19.6 17.0	12.8 18.3	38.1 33.0	16.3 17.9	3.2 2.6	0.4454
Fresh cheeses	B D	11.5 11.9	20.2 16.7	21.2 22.1	37.5 41.0	8.3 8.0	1.3 0.3	0.6004
Cheeses	B D	5.1 6.7	13.8 12.2	20.8 17.6	45.2 44.9	11.5 14.1	3.5 4.5	0.2293
Cold meats	B D	10.3 9.0	9.9 10.6	20.5 20.5	41.7 42.0	10.6 12.5	7.1 5.4	0.9189
Red meats	B D	12.8 17.9	30.4 23.4	28.8 34.3	26.3 22.8	1.6 1.3	0.0 0.3	0.1745
White meats	B D	7.1 6.1	10.9 12.2	26.3 27.2	53.8 51.9	1.6 1.9	0.3 0.6	0.9940
Fishes	B D	8.7 11.5	40.7 38.5	41.7 33.7	8.7 14.7	0.0 1.3	0.3 0.3	0.1531
Eggs	B D	2.6 1.6	9.6 7.4	26.3 24.0	54.2 55.8	5.4 9.6	1.9 1.6	**0.0022**
Pulses	B D	19.2 25.3	50.0 40.7	17.0 18.9	11.9 13.5	1.9 1.3	0.0 0.3	0.7694
Potatoes	B D	4.8 5.1	18.6 10.3	26.9 26.6	44.2 51.6	4.5 6.1	1.0 0.3	**0.0004**
Fruits	B D	0.6 1.6	5.8 7.1	11.9 10.3	34.9 33.7	31.1 30.1	15.7 17.3	0.6880
Vegetables	B D	0.6 1.0	2.6 3.8	9.0 7.4	32.1 33.3	28.5 27.6	27.2 26.9	0.4241
Fast foods	B D	25.6 41.7	57.4 42.0	10.6 11.5	6.1 4.2	0.0 0.6	0.3 0.0	**0.0001**
Fried foods	B D	6.7 9.3	28.5 23.1	29.2 29.2	33.3 36.5	1.6 0.0	0.6 1.9	0.5751
Sweets	B D	5.4 7.4	19.9 16.0	16.7 15.4	38.1 34.0	13.8 16.0	6.1 11.2	**0.0241**
Instant soups	B D	74.7 78.2	16.0 15.1	5.1 3.8	2.6 2.6	1.3 0.3	0.3 0.0	**0.0247**
Tinned meats	B D	73.1 68.3	20.2 19.2	5.1 9.3	1.0 2.6	0.3 0.6	0.3 0.0	**0.0004**
Tinned vegetables	B D	23.7 27.9	27.6 20.8	23.1 22.1	23.1 23.4	2.2 4.2	0.3 1.6	0.2190

B—before confinement, D—during confinement, significant differences are marked in bold.

**Table 5:** The frequency of the consumption of selected beverages before and during lockdown (%).

**Beverage**	**Time**	**Never**	**1–3 Times a Month**	**Once a Week**	**Few Times a Week**	**Once a Day**	**Few Times a Day**	***p*** **-Value**
Fruit juices	B	25.0	27.9	16.0	20.5	7.7	2.9	0.5520
	D	33.7	19.6	15.7	18.3	7.4	5.4	
Vegetable juices	B	45.8	30.1	9.3	10.9	2.9	1.0	0.4047
	D	55.4	19.2	9.6	10.3	3.5	1.9	
Hot beverages	B	1.9	3.2	1.3	5.1	16.0	72.4	0.1500
	D	1.3	3.5	1.6	7.1	16.3	70.2	
Sweetened beverages	B	44.6	29.2	11.9	10.3	2.9	1.3	0.7253
	D	49.0	24.4	11.2	11.2	2.2	1.9	
Energy drinks	B	78.5	14.7	1.6	4.2	0.3	0.6	**0.0150**
	D	85.3	8.3	3.2	1.9	0.6	0.6	
Mineral water	B	0.0	4.8	2.6	10.3	13.1	66.0	0.6431
	D	5.4	3.2	2.2	8.0	11.5	69.6	
Alcohols	B	21.8	37.8	19.6	16.0	3.8	1.0	**0.0031**
	D	25.6	26.0	19.9	22.2	5.4	1.0	

B—before confinement, D—during confinement, significant differences are marked in bold.

**Table 6:** Portion sizes per day before and during confinement (%).

Food Product	Time	0 Serving	0.5 Serving	1 Serving	2 Servings	3 Servings	4 Servings	5 Servings	*p*-Value
White bread	B	7.4	28.8	23.4	17.6	1.9	10.9	9.9	**0.0400**
	D	6.4	34.9	23.4	13.8	4.2	7.1	10.3	
Wholemeal bread	B	16.7	41.7	20.8	12.2	1.9	2.6	4.2	0.1155
	D	20.8	38.5	21.5	11.9	1.0	4.2	2.2	
White rice, white pasta	B	5.4	71.8	19.9	1.9	0.0	1.0	0.0	0.3913
	D	6.4	69.9	19.2	2.9	1.0	0.6	0.0	
Buckwheat, oats	B	9.6	68.3	16.0	3.2	1.0	1.3	0.6	0.7467
	D	11.5	64.7	16.7	5.1	0.0	1.0	1.0	
Butter	B	13.1	31.7	19.9	16.0	1.6	8.3	9.3	0.1506
	D	10.6	34.3	22.1	17.0	2.2	6.1	7.7	
Lard	B	74.0	22.8	2.2	0.6	0.0	0.3	0.0	0.5520
	D	76.6	18.6	3.5	0.6	0.3	0.0	0.3	
Oils or margarines	B	20.8	47.8	18.9	7.1	2.2	1.3	1.9	0.5037
	D	18.6	46.5	22.8	7.7	2.2	1.6	0.6	
Milk	B	16.7	40.4	26.3	10.3	0.3	3.5	2.6	0.6404
	D	16.7	39.1	27.9	9.0	0.3	3.2	3.8	
Fermented milk	B	10.3	62.2	19.6	5.8	0.6	1.3	0.3	0.4484
	D	11.2	62.2	19.9	4.5	1.0	1.0	0.3	
Fresh cheeses	B	11.9	59.3	20.5	6.1	1.0	1.0	0.3	0.0960
	D	11.9	60.9	22.4	3.8	0.3	0.6	0.0	
Cheeses	B	5.4	53.2	28.2	7.4	2.2	1.6	1.9	0.7206
	D	6.7	51.3	28.5	8.0	1.3	1.9	2.2	
Cold meats	B	10.3	49.0	25.0	9.0	1.0	3.8	1.9	0.8761
	D	9.3	49.7	25.3	9.0	2.2	3.2	1.3	
Red meats	B	13.1	74.0	11.5	0.6	0.3	0.3	0.0	**0.0199**
	D	18.6	68.9	11.5	1.0	0.0	0.0	0.0	
White meats	B	7.1	66.3	22.8	2.9	0.6	0.0	0.3	0.4011
	D	6.4	68.6	22.8	1.3	0.6	0.0	0.3	
Fishes	B	8.7	87.5	2.9	1.0	0.0	0.0	0.0	0.7827
	D	11.9	79.8	7.7	0.6	0.0	0.0	0.0	
Eggs	B	2.9	55.1	31.4	7.4	1.3	1.3	0.6	0.2399
	D	1.9	47.4	40.7	8.0	1.0	0.0	1.0	
Pulses	B	19.2	74.0	5.4	1.3	0.0	0.0	0.0	0.2129
	D	25.3	67.0	5.4	2.2	0.0	0.0	0.0	
Potatoes	B	4.8	63.1	25.6	4.8	0.3	0.3	1.0	0.3020
	D	5.1	58.3	30.4	4.8	1.0	0.3	5.1	
Fruits	B	0.6	36.9	29.5	19.2	4.2	4.5	5.1	0.6376
	D	1.6	33.0	35.6	17.0	2.2	5.1	5.4	
Vegetables	B	0.6	31.1	27.6	18.6	3.8	7.4	10.9	0.4464
	D	1.0	34.0	26.6	16.3	4.2	8.3	9.6	
Fast foods	B	26.0	67.6	6.1	0.3	0.0	0.0	0.0	**<0.0001**
	D	41.7	54.8	2.9	0.3	0.3	0.0	0.0	
Fried foods	B	7.1	75.6	14.4	1.9	0.6	0.3	0.0	0.2618
	D	9.3	73.7	15.1	1.0	1.0	0.0	0.0	
Sweets	B	5.8	66.0	19.2	4.5	1.0	1.6	1.9	**0.0029**
	D	7.4	58.0	18.9	7.7	1.0	3.5	3.5	
Instant soups	B	74.7	23.4	1.0	0.6	0.3	0.0	0.0	**0.0283**
	D	78.2	20.8	1.0	0.0	0.0	0.0	0.0	
Tinned meats	B	73.1	25.6	1.3	0.0	0.0	0.0	0.0	**0.0390**
	D	68.6	30.8	0.3	0.0	0.3	0.0	0.0	
Tinned vegetables	BD	23.727.9	66.759.6	7.79.6	1.91.9	0.00.0	0.00.0	0.01.0	0.8251

B—before confinement, D—during confinement, significant differences are marked in bold.

**Table 7:** Portion sizes per day before and during confinement (%).

Beverage	Time	0 Serving	0.5 Serving	1 Serving	2 Servings	3 Servings	4 Servings	5 Servings	*p*-Value
Fruit juices	B	25.6	55.8	13.5	2.2	0.6	1.3	1.0	0.4344
	D	34.0	46.8	13.5	2.2	0.0	1.0	2.6	
Vegetable juices	B	46.5	45.8	5.8	0.6	0.3	0.6	0.3	0.0749
	D	55.8	34.3	7.4	1.3	0.3	0.6	0.3	
Hot beverages	B	1.9	7.4	10.6	29.5	1.6	10.9	38.1	0.1237
	D	1.3	8.0	12.2	30.1	1.3	13.1	34.0	
Sweetened beverages	B	44.6	44.9	6.1	3.2	0.3	0.3	0.6	**0.0254**
	D	49.4	42.0	5.8	1.3	0.0	1.6	0.0	
Energy drinks	B	78.5	18.3	2.2	0.3	0.0	0.0	0.6	**0.0008**
	D	85.3	13.5	0.3	0.0	0.0	0.6	0.3	
Mineral water	B	3.2	11.2	16.7	19.9	1.0	11.5	36.5	0.6482
	D	5.8	10.9	12.2	21.2	0.6	12.2	37.2	
Alcohol	B	22.1	59.9	13.8	2.9	0.3	0.6	0.3	0.1859
	D	25.6	51.9	16.7	3.5	1.3	0.3	0.6	

B—before confinement, D—during confinement, significant differences are marked bold.
